# Notes and News

**Published:** 1900-12-15

**Authors:** 


### NOTES AND NEWS.

The late Mr. James Leach has bequeathed £1,000 to the Rochdale Infirmary.

The Special Commission appointed by the French Minister of Public Instruction on the subject of Tuberculosis more especially, has finished its deliberations and made its recommendations very complete. Preventive and curative measures are recommended, which include and go beyond the scope of the establishment of sanatoria.

The new wing at the Stockport Infirmary, which has been formally opened, constitutes the Jubilee Memorial of Stockport. It comprises two wards, an operating theatre, anæsthetic, surgeons', and recovery rooms; new nurses quarters, recreation and day rooms for patients, laundry, mortuary, post-mortem rooms, and offices. The cost has been £12,000.

Wards to accommodate 120 imbeciles have been opened at the Leeds Workhouse Infirmary. The wards are situated in two blocks of two storeys each, with an administration block in the centre, all being connected by corridors, which can be utilised as day rooms. Outside staircases in case of fire have been provided The provision of these wards will admit of the separation of epileptic patients from imbeciles. The cost of the building has been £16,500.

The last report from the Imperial Yeomanry Hospital shows that there were 19 officers and 566 non-commissioned officers and men in the hospital in Deelfontein, of whom 12 officers and 455 men belonged to the Imperial Yeomanry, and that 59 Yeomen were expected. At Pretoria, 36 officers and 259 men were under treatment in the hospital, amongst which there were 40 cases of enteric fever which was apparently on the increase. At the Imperial Yeomanry Branch Hospital at McKenzie's Farm, Maitland, there remained in the hospital 117 patients, of whom 111 were Yeomen. The commandants of the Imperial Yeomanry Field Hospital and Bearer Company report that they are still at Pretoria waiting marching orders, adding that the heavy rains and severe thunderstorms which were raging would probably necessitate their remaining stationary for some time as the surrounding country had practically become a swamp.

There is still a considerable amount of plague in India, chiefly in the Bombay Presidency, Poona, Calcutta, Bengal, and Mysore. In Mauritius fresh cases continue to present themselves. In South Africa the disease is apparently arrested.

The New Medical Bill in New South Wales is practically accepted. It aims at the protection of the public from quackery by the registration of all legally-qualified practitioners, and the punishment of all those who assume titles they have no legal right to or who pose as legally-qualified practitioners.

Through the will of the late Sir Henry Page Turner Barron, formerly resident British Minister at Stuttgart, the Prince of Wales's Hospital Fund becomes possessed of £3,000. Sir Henry has bequeathed £30,000 to the Irish charities.

A meeting was recently held at Bristol, under the auspices of the Gloucester, Somerset, and Wilts branch of the National Association for the Prevention of Consumption. The Lord Mayor of Bristol presided over the meeting, which was representative of the three counties. A resolution was passed in favour of starting the scheme to establish and maintain a sanatorium for these counties. It is proposed to place the institution on the best possible site, and to provide a Resident Medical Superintendent. Sixty patients are to be provided for at a proposed cost of £15,000 for the building, and a yearly expenditure of £4,000. The committee hope to receive help from County Councils, Town Councils, and clubs and societies.

This Christmas we are presenting to our readers two supplements. Our Appeal Supplement tells in narrative form the story of the sick, within the hospitals and in the home, at Christmas time. It is the production of many writers, all eye witnesses of the facts they relate, and the tales they tell bear an unmistakable stamp of truth. Our second supplement consists of five beautiful plates illustrating Christmas scenes within the hospitals, which give examples of what is accomplished by way of decoration by the busy medical and nursing staff. Like the literary pictures in the Appeal Supplement they are taken from life.

The War Office has applied to the authorities of Glasgow University for qualified surgeons for South Africa, to take the places of those who are returning. The appointments will be for twelve months.

The St. John's Ambulance Association has a large following amongst the employes of the Great Western Railway. Last week upwards of 200 medallions, vouchers, and certificates were presented to members of the staff by the Countess of Cork and Orrery. Over 5,500 members of the staff now possess the first-aid certificate of the St. John's Ambulance Association. Of these, 2,200 have passed the second examination and 1,100 the third. During the past twelve months upwards of 1,000 cases have been treated by duly-qualified members of the company's staff. Fifty-eight Great Western Railway Ambulance Volunteers have been on service in South Africa, and sixteen of these were specially selected for duty with the Imperial Yeomanry Hospital at Deelfontein. Five deaths have been reported by the War Office amongst the Great Western Railway Volunteers. Earl Cawdor, the chairman of the Great Western Railway Company, presided at the meeting.

